# The relationship between the level of μ-opioid receptor (μORs) and postoperative analgesic use in patients undergoing septoplasty: a prospective randomized controlled trial

**DOI:** 10.1186/s12871-020-01138-z

**Published:** 2020-09-08

**Authors:** Muzaffer Gencer, Ayşe Yeşim Göçmen

**Affiliations:** 1 Department of Anesthesia, Istinye University Medical Faculty, Istanbul, Turkey; 2 Department of Biochemistry, Bozok University Medical Faculty, Yozgat, Turkey

**Keywords:** Analgesic agent, μ-Opioid receptor (μORs), Septoplasty, Tramadol

## Abstract

**Background:**

In this study, the μ-Opioid receptor activity was assessed pre-operatively for its association with postoperative pain level and second analgesic requirement in patients undergoing septoplasty.

**Methods:**

In our prospective study, 120 adult patients underwent septoplasty from June 2015 to January 2019 were randomly divided into 2 pre-operative groups. The first group (*n* = 60) was patients given tramadol (1–2 mg/kg) for post-operative analgesia, and the second group (control group) (*n* = 60) was initially prescribed only fentanyl (1 μg/ kg-i.v.) in the induction. Acetaminophen with codeine analgesic 325/30 mg (p.o.) was used as an rescue painkiller in the post-operative period. The μ-Opioid receptor activity was investigated in pre-operative blood samples and compared to post-operative pain level and time required for second round of analgesic administration. The visual analogue score (VAS) was used to evaluate the post-operative pain degree (0 no pain; 10 worst pain). The patients’ post-operative VAS scores were evaluated upon arrival to recovery room, and at the 1st, 3rd, 7th, 10th, and 24th hour post-operative period.

**Results:**

Demographic data and peri-operative variables were similar in both study group (*p* < 0.05).There was no significant difference between the receptor levels in both groups and the mean receptor level was 200.94 ± 15.34 pg/mL (max:489.92 ± 22.36 pg/mL, min: 94.56 ± 11.23 pg/mL).In patients who used tramadol as the levels of μ-Opioid receptors increased, VAS scores of patients and second analgesic use decreased in post-operative period.The VAS scores in patients with higher receptor levels were lower in the recovery room (*p* < 0.05), 1st (*p* < 0.05) and 3rd hours (*p* < 0.05).The VAS scores were lower in the tramadol group compared to the control group (*p* < 0.05).Number of secondary analgesic requirement was significantly lower in patients of the tramadol group with higher receptor levels compared to the ones with lower receptor (*p* < 0.05) for arrival at the recovery room and 1st hour. Patients in the tramadol group needed a second pain killer much later than patients in the control group.

**Conclusions:**

Our study demonstrates that patients with higher μOR levels have a higher efficacy of opioid analgesic agents and an lesser need for additional analgesic agents.

**Trial registration:**

This trial was registered retrospectively (The ACTRN: ACTRN12619001652167, registration date: 26/11/2019).

## Background

Nasal septal surgery is one of the most common operations in otorhinolaryngology; alone or in combination with other procedures, such as inferior turbinoplasty, endoscopic sinus surgery, and rhinoplasty. Nasal septal surgery performed by an otolaryngologists may cause severe pain post-operatively. In the post-surgery term, patients usually suffer from severe pain for several days and the pain slowly decreases over the following 4 days [1]. *Non*-*steroidal* anti-inflammatory *drugs* (NSAIDs)*,* acetaminophen, and opioid analgesics can be used as medications for post-surgical pain control. Different methods and techniques have been used to reduce pain, including improved intraoperative anesthetic pain regimens, adjustment of surgical technique, and intra-operative local anesthesia infiltration. Presently, the drugs used in the field of post-operative analgesia are mainly opioids. Opioid analgesics provide significant benefits for relief of moderate-to-severe pain. A number of opioids are available for clinical use such as fentanyl, remifentanil, and tramadol. Tramadol is commonly used as an opioid analgesic for post-operative analgesia. Tramadol has important advantages compared to the other opioids including a long duration of action, rapid recovery, and limited hemodynamic and respiratory depressant effects. Tramadol and the metabolite O- desmethyl-tramadol (M1) are agonists of the mu (μ) opioid receptor [2]. Tramadol, a centrally acting analgesic, also stimulates pre-synaptic release of serotonin and inhibits serotonin reuptake. Therefore, tramadol increases inhibitory effects on pain transmission both by opioid and monoaminergic mechanisms [3, 4]. Due to its pharmacological properties, tramadol is a safe drug that has a low risk of drug abuse and dependence, respiratory depression, and cardiovascular side effects unlike other opioids [5].

Opioid receptors are classified as the mu-opioid receptors (MOP-R), kappa-opioid receptors (KOP-R) and delta-opioid receptors (DOP-R) and can be heterogeneous upon multimerization [6].

The pharmacological effects of opioid analgesics are derived from their complex interactions with three opioid receptor types (mu, delta, and kappa). The mu opioid receptor gene (*OPRM1*) (opioid receptor, mu 1) produces a receptor (the MOP-r) that is a site of action for commonly used opioid analgesics [7]. μ-Opioid receptors (μORs) are the major receptors that mediate the analgesic effects of opioids. (μ)-Opioid receptor agonists such as fentanyl, remifentanil, and morphine are the gold standard treatment for severe pain. However, opioid analgesic agents are prone to abuse due to their highly addictive effect and their use may cause undesirable side-effects including respiratory distress, sedation, locomotor activity, constipation, narcotic addiction, and tolerance. The use of these agents in post-operative analgesia is limited due to mechanisms such as respiratory depression, sedation, tolerance and dependence [8]. μ-Opioid receptors bind to G proteins, and their activity in periaqueductal gray matter and brainstem is associated with analgesic effects [9].

In a recent study, the researchers revealed that polymorphism in the μ-Opioid receptor gene may cause a change in the patient’s pain threshold and susceptibility to opioid drugs [10]. When the current literature is reviewed, there is limited number of studies related to the relationship between opioid agents and the μ-Opioid receptor level.

In our study, we aimed to investigate the relationship between the μ-Opioid receptor activities with post-operative pain level and second analgesic administration requirement in nasal septal surgery patients.

## Methods

This study was a randomized, double-blind, and prospective trial. Between June 2015 and January 2019, 120 adult patients underwent septoplasty at Otorhinolaryngology Clinic of Bozok University Research Hospital were included to the study. The approval of the Ethics Committee was obtained (date: May 25, 2015, number: 25/12). This trial was registered retrospectively (The ACTRN: ACTRN12619001652167, registration date: 26/11/2019).

The informed consents were obtained from all patients and followed the guidelines of Helsinki. In the operation room, all patients were randomly classified into two groups by using a computer-generated randomization table with an allocation ratio of 1:1. The randomization table was obtained from the website http://www.randomization.com. The randomization was performed by an anesthesiologist who was not involved in the anesthetic management. Intraoperative and post-operative data was collected by an anesthesiologist and *anesthetic nurses* who did not participate in the study. For post-operative analgesia, the first group (*n* = 60) used tramadol and the second group (*n* = 60) were given fentanyl in the induction initially. In both groups, fentanyl (1 μg/ kg-i.v.), propofol (2–3 mg/ kg), and muscle relaxant (rocuronium bromide 0.6 mg/ kg) were administered to all patients for induction. After endotracheal intubation, the rest of the anesthesia procedure was maintained with 2–3% sevoflurane. Sixty percent NO_2_ in 40% O_2_ was delivered to the patients in both groups. Although at the end of the surgery to first group patients was given tramadol (1–2 mg/ kg) for post-operative analgesia, no agent was given to the control group for post-operative analgesia. The patients in control group received same amount of placebo instead of tramadol *100 mg* vial (50 mg/ml, 2 mL). The medications given intravenously to each group before awakening were performed by the *Anesthesia* Care *Team.* Acetaminophen with codeine analgesic 325/30 mg (p.o.) was used as an additional analgesic agent in the post-operative period.

The inclusion criteria for the study consisted of patients between the age of 18–45 years, who were categorized as I and II according to the American Society of Anesthesiology physical status classification and scheduled for elective surgery for septoplasty operation under general anesthesia. The exclusion criteria consisted of the patients who had electrocardiogram (ECG) changes, receiving opioids for chronic pain, additional nasal pathologies and thus receiving additional surgical intervention, and history of allergies to local anesthetics, pregnancy, renal insufficiency, cognitive dysfunction and refusal of participation to the study**.**

All patients were operated by the same surgical team with similar techniques under general anesthesia by using the classic septoplasty operation technique including the correction of a deviated septum, classic submucosal resection, traditional septoplasty, and open techniques [11]. Since the genetic analysis of the samples was not available in our institute, venous blood samples were obtained from patients for research to determine the μ-Opioid receptors activities in the pre-operative period. The sera were transferred into unused cover tubes. The tubes were stored at − 20 °C in the deep-freezer and analyzed for μ-Opioid receptors levels using an Olympus AU 600 auto-analyzer (Olympus Optical Co., Japan) using Randox kits.

All the patients’ vital signs were monitored during the operation. In all patients, the changes of mean arterial pressure, heart rate and Ramsay Sedation Scales (RASS) were measured at predetermined time points as arrival to the recovery room, and at the 1st, 3rd, 7th, 10th, and 24th hours in post-operative period.

To determine the level of post-operative pain, a continuous 10 cm visual analog scale (VAS), was used. On the scale, 0 indicated ‘no pain’, and 10 indicated ‘severe pain’. The patients were asked to mark their pain at different times on the scale, and the results were recorded. First measurements were made on arrival to the recovery room in postoperative period, and they were repeated at the 1st, 3rd, 7th, 10th, and 24th hours. When VAS pain scale was evaluated at postoperative 1st hour (in addition to the patient’s level of consciousness), clinical signs and vital signs were also evaluated. At the times when the pain was severe (VAS ≥ 4), the patients were given upon arrival to the recovery room: Acetaminophen 1 g (10 mg/mL, 100 mL) intravenously due to difficult peroral intake, at other time points: Acetaminophen with codeine analgesic 325/30 mg perorally as rescue analgesic, and both timing and amount of analgesics used were recorded. The relations between μ-Opioid receptors level and VAS pain scale and second analgesic need was investigated in patients. The primary outcome was the postoperative pain level difference in relation with pre-operative μORs level. The secondary outcomes were the needed rescue analgesic agent (Acetaminophen with codeine analgesic 325/30 mg. per-oral) timing and amount, the changes of mean arterial pressure, heart rate, the degree of sedation of the patients, incidence of postoperative nause and vomiting in post-operative period.

### Statistical analysis

Sample size calculation were performed with a power analysis based on data from a previous study [12]. In this study, which included a total of 96 patients, the relationship between Human mu opioid receptor gene A118G polymorphism and efficacy of a combination of tramadol and acetaminophen was investigated in painful neuropathy. In the study, the researchers revealed that Human mu opioid receptor gene A118G polymorphism decreased analgesic efficacy of opioid agents in pain control. Power estimation analysis suggested that 53 patients per group with a power of 80% (1-β error = 0.80), considering a type I error of 0.05 (α error = 0.05). To compensate for unexpected losses, recruitment was increased by 20%. The data were analyzed using the SPSS 21.0 software package. The number, mean and standard deviations of the demographic variables were tabulated, and student t test was used to compare the groups. ANOVA test (two ways classification with repeated measures) was used for statistical analysis of VAS values. A *p*-value of less than 0.05 was accepted as statistically significant.

## Results

**Fig. 1:**
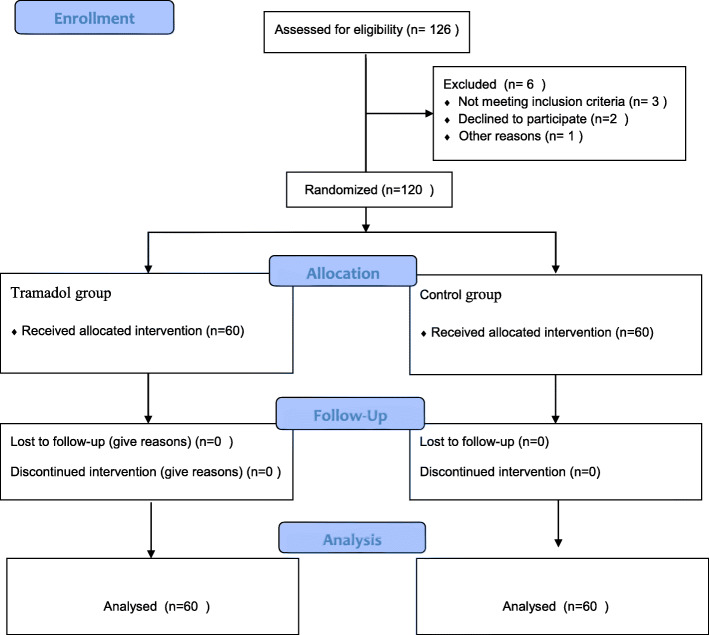
Flow chart of the study

One hundred twenty adult patients underwent septoplasty were randomly selected for two groups. There were 52 female and 68 male patients (ranged from 18 to 45 years of age). One hundred twenty-six patients were enrolled randomly and 120 were included in the analysis. Six patients were excluded the study because they did not agree to participate. A consort flow diagram of the study is shown in Fig. 1.

The two groups were comparable with respect to age, gender, American Society of Anesthesiologists Scale (ASA), body mass index (BMI), surgical time, and anesthesia time. There was no statistically significant difference between the two groups in terms of demographic data and perioperative variables (Table 1).

**Table 1:** Demographic data and perioperative variables

	Tramadol (*n* = 60)	Control (*n* = 60)	^*^P value
Age (yr)	28.4 ± 10.02	32.26 ± 11.78	0.352
Sex (F/M)	24/36	28/32	0.466
ASA Score I/II	21/39	31/29	0.231
BMI (kg/m^2^)	23.4 ± 3.0	25.3 ± 5.0	0.285
Duration of surgery (min)	74.44 ± 23.81	80.48 ± 25.14	0.406
Duration of anesthesia (min)	82.91 ± 25.75	85.38 ± 29.31	0.763

Data are expressed as number of patients and mean ± SD. *ASA* American society of Anesthesiologists, *BMI* Body Mass Index, *F* Female, *M* Male.* Student t test, *p* > 0.05

In tramadol group, compared to patients with a μORs level of 200.94 ± 15.34 pg/mL-489.92 ± 22.36 pg/mL and patients with a μORs level of 94.56 ± 11.23 pg/mL-200.94 ± 15.34 pg/mL, patients with a higher receptor level were less painful and the VAS scores were lower at the recovery room, (*p* < 0.001), 1st hour (*p* < 0.001), 3rd hour (*p* < 0.05), 7th hour (*p* < 0.05), 10th hour (*p* < 0.05) in post-operative period. In the control group, while the VAS scores in patients with higher receptor levels (range: 200.94 ± 15.34–489.92 ± 22.36 pg/mL) were lower in the recovery room, (*p* < 0.05), 1st (*p* < 0.05) and 3rd hours (*p* < 0.05), there was no significant difference in other time points. Additionally, compared to the control group, the VAS scores were significantly lower in the tramadol group with both receptor levels 200.94 ± 15.34–489.92 ± 22.36 pg/mL (*p* values were < 0.001 for arrival at the recovery room and 1st hour, p values were < 0.05 for 3rd, 7th and 10th hours, not significant for 24th hour) and receptor levels 94.56 ± 11.23–200.94 ± 15.34 pg/mL (p values were < 0.01 for arrival at the recovery room and 1st hour, p values were < 0.05 for 3rd and 7th hour, not significant for 10th and 24th hours). We commented these data as follow: the severity of pain of post septoplasty in study group patients was less observed in the tramadol group than the control group at post-operative arrival, 1st, 3rd, 7th, and, 10th hours. Moreover, the effect of time (post-operative hours) on VAS values was significant in both the tramadol group and the control group (Table 2). The second analgesic agent requirement was significantly different between tramadol group and control group. The patients in the tramadol group required a second painkiller at a later hours and less amount than the control group who only received fentanyl in induction.

**Table 2:** Visual analogue *scale* (*VAS*) and second analgesic use between the groups

	Arriva	1st h	3rd h	7th h	10th h	24th h
μORs level: **200.94–489.92 pg/mL**						
Group T (*n* = 32) VAS score	1	1	2	1	1	1
	(0–2)^b^	(0–2)	(1–3)	(0–2)	(0–1)	(0–1)
R. Analgesic^a^	0	0	1	0	0	0
Group C (*n* = 31) VAS score	5	4	3	2	2	1
	(3–8)^b^	(3–5)	(2–5)	(1–3)	(1–2)	(0–2)
R.Analgesic	2	0	0	0	1	0
μORs level: **94.56–200.94 pg/mL**						
Group T (*n* = 28) VAS score	3	3	3	2	2	1
	(2–5)^b^	(1–5)	(1–5)	(1–3)	(1–2)	(0–1)
R. Analgesic	1	1	1	1	1	0
Group C (*n* = 29) VAS score	6	5	4	3	2	1
	(4–8)^b^	(3–7)	(3–5)	(1–3)	(1–2)	(0–2)
R. Analgesic	2	2	1	1	1	0

^a^R. Analgesic: Rescue analgesic use

^b^Min-Max values. Group T: Tramadol group; Group C: Control Group. VAS scores were expressed in median. Acetaminophen 1 g (10 mg/ml,100 ml) intravenously was given at arrival to the recovery room as a rescue analgesic, Acetaminophen with codeine analgesic 325/ 30 mg (p.o) was given at other time points as a rescue analgesic; 0: analgesic was not given; 1: one dose was given; 2: two doses were given

Compared to patients with μORs level: 200.94 ± 15.34–489.92 ± 22.36 pg/mL and patients with μORs level: 94.56 ± 11.23–200.94 ± 15.34 pg/mL; number of secondary analgesic requirement was significantly lower in patients of the tramadol group with higher receptor levels compared to the ones with lower receptor (*p* values were < 0.05 for arrival at the recovery room and 1st hour whereas not significant for the other time points). In the control group, when the patients whose μORs level were above the average (200.94 ± 15.34 pg/mL) and those below the mean were compared, number of secondary analgesic use was higher in patients with μORs level: 94.56 ± 11.23–200.94 ± 15.34 pg/mL (*p* values were < 0.05 for arrival at the recovery room and 1st hour whereas not significant for the other time points) (Table 2). These results suggest opioids effect patients more with high receptor levels and therefore; patients felt lower pain in the postoperative period. *VAS* and a second analgesic need in both the tramadol group and the control group are shown in Table 2.

Mean arterial pressure was significantly lower in the 1st and 3rd hours in post-operative period in the tramadol group compared to the control group**.** Similarly, the heart rate of patients was higher in the control group than in the tramadol group at the time of arrival in the recovery room and post-operative 1st and 3rd hours (Table 3).

**Table 3:** The changes of mean arterial pressure and heart rate at different time points

	Tramadol group (***n*** = 60)	Control group (***n*** = 60)	***p***-value
**Arterial pressure** (Mean ± SD)			
Arrival	116.54 ± 15.92	124.91 ± 11.06	0.354
1st h	98.54 ± 15.88	106.88 ± 11.66	0.048^**^
3rd h	91.78 ± 3.36	93.62 ± 2.12	0.030^**^
7th h	83.34 ± 10.06	84.20 ± 10.96	0.846
10th h	78.20 ± 7.62	77.00 ± 6.82	0.636
24th h	71.54 ± 3.34	72.76 ± 2.46	0.172
**Heart rate** (Mean ± SD)			
Arrival	88.03 ± 5.22	104.14 ± 5.82	0.001*
1st h	86.51 ± 5.15	102.73 ± 5.78	0.001*
3rd h	85.93 ± 5.02	100.86 ± 5.86	0.001*
7th h	79.12 ± 3.20	92.14 ± 2.60	0.192
10th h	78.50 ± 2.32	86.58 ± 1.82	0.146
24th h	72.84 ± 4.93	94.34 ± 5.74	0.318

*SD* Standard deviation, *h* hour. Student t test * *p* < 0.01, **p < 0.05

Ramsay Sedation Scale (RASS) scores were similar in both groups. However, patients in the control group were observed to be more agitated at the post-operative 3rd and 7th hour time points, but it did not reach to level of clinical significance. RASS of the patients in both study groups are shown in Table 4.

**Table 4:** The comparison of Ramsay sedation scores of the tramadol and the control groups

Time points	Tramadol (*n* = 60)	Control (*n* = 60)	*p* values
Arrival	3 (2–3)	3 (1–3)	0.452
1th hour	2 (2–2)	2 (1–2)	0.406
3th hour	2 (2–2)	1 (1–2)	0.132
7th hour	2 (2–2)	1 (1–1)	0.095
10th hour	2 (2–2)	2 (1–2)	0.314
24th hour	2 (2–2))	2 (1–2)	0.324

Data are expressed as median

Comparison of the incidence of vomiting between the groups did not show any significant difference during post-operative period. Five patients in the tramadol group and three patients in the control group had nausea and vomiting in the recovery room during the post-operative period (*p* = 0.464). Three patients developed respiratory distress in the tramadol group, and two patients were *reintubated* due to decrease in peripheral oxygen saturation (SpO_2_) in the control group. Only 3 patients had bleeding as postoperative complications.

## Discussion

This is the first prospective study investigating the relationship between μ-opioid receptor level and post-operative pain and analgesic use. As the level of the μ-Opioid receptors increased, the effect of opioid analgesics such as the tramadol increased in study group.

After elective rhinologic surgery, pain is prominent in the first 3 days, but rapidly decreases in the days that follow [13]. Patients who undergo septoplasty operations will experience the most pain within the first 24 h, and patients often need additional analgesics during this period. The pain that occurs in the post-operative period is mostly associated with surgical trauma and the release of pain mediators into the circulation [1]. Controlling pain during the post-operative period reduces pain-related anxiety in the patient and thus, prevents the development of a cascade that may have negative consequences for the patient [14]. Low pain level of the patient will speed up recovery, provide a comfortable process, and minimize the cost [15]. It is beneficial for the patient to apply a local anesthetic agent to the surgical area during the surgery as it causes decreased post-operative pain scores and additional analgesic requirements [16]. In a recent study, the addition of a local anesthetic agent to the nasal packs after septal surgery has been shown to have positive effects in reducing post-operative pain within the first 12 h [17].

In our study, we investigated the relationship between μ-Opioid receptor level and opioid analgesics and evaluated with post-operative pain and analgesic use. However, the current studies revealed that the μ-Opioid receptors are not only associated with pain, but are also closely related to some tumor cells. Recently μ-opioid receptors have been shown to be in many cancer cell lines including non-small cell lung cancer, breast cancer, adenocarcinoma, and gastric carcinoma [18, 19]. The current studies have revealed that MOR expression correlated with, tumor aggressiveness, progression-free survival, and survival [20]. Levins KJ and colleagues [21] reported that there are the relationship between some tumor cells in the body and the anesthetic technique and μ-Opioid receptors. In their study, they emphasized that tumor MOR expression is a key difference and that this difference has prognostic importance in most types of cancer. It is possible that difference in μ-Opioid receptors may be caused by the interaction between opioid analgesic use (morphine) and the OPRM1 gene causing an increase in MOR expression. They reported a relationship between MOR expression and anesthetic technique and suggested that the use of regional anesthetic techniques and total intravenous anesthesia could be more appropriate anesthesia methods in oncoanesthesia.

Steroids such as methylprednisolone are used due to anti-inflammatory and immunosuppressive effects in addition to opioid analgesics for post-operative pain [22]. Their effects take place by altering the gene expression with specific intracellular receptor action; this leads to the blockage of the formation of certain substances, and the acceleration of the production of others. As a result, there is reduced edema and fibrosis during healing [23]. Dexamethasone may reduce inflammation at the surgery site by reducing release of inflammatory mediators into the circulation [24]. Dexamethasone significantly reduced the μ-opioid receptor binding in the adrenal cortex and affects differently opioid receptor binding in the hypothalamus and pituitary gland [25].

In addition to opioid analgesics and steroids, some drugs may also be used in post-operative pain. Kim et al. [26] revealed that oral administration of 150 mg of pregabalin twice in the early postoperative period is an effective and safe option in early postoperative pain relief in patients undergoing septoplasty. Non-opioid analgesics and NSAIs are commonly used drugs to reduce pain and inflammation after surgery. However, the use of these drugs by clinicians is limited, as excessive use of these agents can lead to gastrointestinal damage, which can be serious enough to cause bleeding.

Although opioid analgesics have side effects, they are commonly used agents for post-operative analgesia. Tramadol has been used frequently in recent decades and opioid drugs show their analgesic effects by affecting μ-Opioid receptors. One of the ways under the analgesic effect of tramadol is the affinity to μ-opioid receptors. It binds stronger to μ-Opioid receptors than the δ-Opioid or κ-Opioid receptors [2]. Another factor contributing to the analgesic effect of tramadol is the inhibition of the reuptake of monoamines such as norepinephrin and 5-Hydrositriptamin, which play a role in the transmission of pain in the central nervous system (CNS) [15]. Agents such as carbamazepine and cimetidine, which induce hepatic enzyme decreases the effect of tramadol. It has been shown in studies that the dose of tramadol should be increased when used with such drugs [27]. Tramadol’s analgesic effect lasts 2–3 times longer than fentanyl and provides analgesia for about 7–8 h [2]. Fentanyl is a synthetic, lipophilic phenylpiperidine opioid agonist, and produces its potent analgesic effects for the treatment of moderate to severe pain via activation of the μORs with low affinity for delta and kappa opioid receptors. Unlike tramadol, which is a centrally acting weak μ opioid agonist, fentanyl is a highly efficacious agonist at the μORs, and it has a faster onset, much shorter duration of analgesic action, and higher analgesic potency compared to tramadol [28]. Undesirable side effects associated with opioid analgesic use can be seen, and opioid misuse, abuse, dependence, addiction, and overdose deaths are a major cause of concern for clinicians [8]. Since tramadol is a weak μ-opioid agonist that affects the centrally, its tolerability is higher compared to fentanyl, and adverse side effects such as respiratory depression, constipation, abuse, dependence and abuse potential are lower than other opioids [29]. Acetaminophen used as rescue analgesic in the study is a centrally acting analgesic that appears to relieve pain through both spinal and supraspinal levels. The combination of tramadol and acetaminophen may provide pain relief with synergistic effect in a 1: 8 ratio through analgesic effect in multiple pathways [30]. Granados-Soto and colleagues showed that tramadol combined with gabapentin showed a synergistic effect in both systemic and spinal administration [31]. Tramadol can cause serotonin syndrome when with serotonin reuptake inhibitors (SSRIs) and tricyclic antidepressant (TCA). A case of serotonin syndrome has been reported in the literature related to sertraline [32].

Endogenous opioids acting by binding to μ-Opioid receptors are likely to interact with hormones released from the hypothalamic-pituitary-adrenal axis in physiological and pathophysiological conditions [33].

There are a few limitations in the study. First, we used the weak μ-Opioid receptor agonist, tramadol in our study to investigate the relationship between the μ-Opioid receptor activities with post-operative pain level and second analgesic administration requirement. In similar studies, more efficient results may be obtained when using other opioid analgesics, which are more potent, highly efficacious agonists at the μORs. Second, genetic analysis of spinal or supraspinal tissue samples could be used for the measurement of mu opioid receptors. However, genetic analysis of samples is not available in our institute, patients’ venous blood samples were used for research to determine the μ-Opioid receptors activities in the pre-operative period. Finally, we included 120 adult patients in the study. Similar studies may be carried out with more participants.

## Conclusions

In this study, we found that the efficacy of opioid analgesic agents was higher and the need for additional analgesics was lower in patients with higher μ-Opioid receptor levels. As the level of the μ-Opioid receptor increased in the study groups, the duration of the second analgesic requirement increased. Patients with a high level of μORs in both study group experienced less analgesic need in the post-operative period. Additionally, Tramadol is a safe and effective opioid analgesic agent that reduces the postoperative pain and it may be effective analgesic agent of choice in septoplasty operations. We recommend the use of opioids such as tramadol in patients with higher opioid receptor levels for more comfortable post-operative periods.

## Data Availability

The datasets used and/or analyzed during the present study are available from the corresponding author on reasonable request.

## Abbreviations

μORs: Mu-opioid receptor
ΚORs: Kappa-opioid receptors (KOP-R)
DOP-R: Delta-opioid receptors (DOP-R)
VAS: Visual analog scale
RASS: The ramsay sedation scale
NE: *Norepinephrine*
5-HT: *5*-*Hidroksitriptofan*
SSRIs: Serotonin reuptake inhibitors
